# Smith c/b ratio and teardrop figure measurements; Can it be used in the follow-up of patients who underwent salter innominate osteotomy?

**DOI:** 10.1097/MD.0000000000035278

**Published:** 2023-09-15

**Authors:** Kadir Ismail Dere, Duran Topak, Ökkeş Bilal, Fatih Doğar, Mustafa Abdullah Özdemir, Burak Kuşcu

**Affiliations:** a Bingol Solhan State Hospital, Clinical of Orthopaedic and Traumatology, Solhan, Bingol, Turkey; b Kahramanmaras Sutcu Imam University, Faculty of Medicine, Department of Orthopaedic and Traumatology, Kahramanmaras, Turkey; c Pazarcik State Hospital, Clinical of Orthopaedic and Traumatology, Pazarcik, Kahramanmaras, Turkey.

**Keywords:** c/b ratio, developmental hip dysplasia, salter innominate osteotomy, teardrop

## Abstract

**Background:**

This study aimed to investigate the effectiveness of radiological parameters used in the follow-up of patients who underwent salter innominate osteotomy (SIO) for the treatment of developmental dysplasia of the hip.

**Methods:**

Acetabular index, c/b ratio, teardrop width, femoral head teardrop distance (TDD), and acetabular teardrop angle were measured on anteroposterior pelvic radiographs of patients who underwent SIO between 2017 and 2020. The patients were divided into 2 groups according to their preoperative Tönnis stage. Twenty-five (51%) hips of 23 patients with Tönnis stage 2 were classified into group 1, and 24 (49%) of 17 patients with Tönnis stages 3 and 4 were classified into group 2. Changes in radiologic parameters over time and between the groups were statistically evaluated.

**Results:**

The study included 49 hips of 40 patients (37 female and 3 male). The age at surgery was 26.53 (18–53) months. After a mean follow-up period of 33.7 ± 12.8 months, there was no statistically significant difference between Groups 1 and 2 in terms of clinical, radiological and femoral head avascular necrosis results (*P* = .591, *P* = 956, *P* = .492). The changes in radiological parameters over time and between groups were statistically significant. (*P* < .001). Only the TDD and c/b ratio were significantly different between groups 1 and 2 (*P* = .002 and *P* < .001, respectively).

**Conclusion:**

In our study, along with acetabular index, the c/b ratio, teardrop width, TDD, and acetabular teardrop angle significantly changed after SIO and could be used as a guide for patient follow-up.

## 1. Introduction

Developmental dysplasia of the hip (DDH) occurs as a result of a developmental defect in the hip joint and refers to a wide range of conditions from subluxation to dislocation. Diagnosis and treatment are important as they are common in childhood and can cause disability if left untreated.^[1]^

DDH can be treated either conservatively or surgically.^[1–4]^ With early diagnosis, treatment is possible with conservative methods such as splinting or pelvic pedal casting after closed reduction.^[2,3]^ However, when the diagnosis is delayed or primary treatment fails, a pelvic reconstruction procedure is often required in addition to open reduction.^[1,5]^ Salter innominate osteotomy (SIO) is the most commonly used reconstructive procedure with favorable results.^[1]^ SIO is based on repositioning of the acetabulum anteriorly, distally, and laterally in the reduced hip.^[1,6–8]^

DDH is a dynamic disease and long-term follow-up is required to evaluate its efficacy.^[1,7,8]^ A number of clinical and radiological classifications have been defined to evaluate the outcome of treatment.^[1,8–11]^ The frequently preferred radiological classification is the Severin classification because it is simple and easy to apply, while clinical classification is often made using the McKay classification.^[11]^ Kalamchi–MacEven classification is used to evaluate femoral head AVN that occur as a result of treatment.^[1]^

In addition, it is possible to make early predictions regarding the prognosis using radiological parameters.^[12,13]^ Radiological parameters are usually evaluated by anteroposterior radiography of the pelvis.^[1]^ Many measurements including the acetabular index (AI), c/b ratio, teardrop measurements, and center edge angle can be obtained in pelvic radiography to evaluate hip development and monitor outcomes after DDH treatment.^[1]^

The aim of the present study was to evaluate the clinical and radiological results of patients who underwent SIO and to investigate the efficacy of AI, c/b ratio, and teardrop radiological measurements in the follow-up of treatment and estimation of prognosis.

## 2. Methods

The files of the patients who underwent SIO for DDH between January 2017 and December 2020 were retrospectively reviewed after obtaining approval from the local ethics committee (date: March 15, 2021, session no: 2021/10, decision no: 01).

The inclusion criteria were as follows: patients between the aged between 18 months and 6 years who underwent SIO due to DDH, had a follow-up period of at least 1 year, had no missing data in their files, and had regular clinical and radiological follow-up. A total of 48 patients were screened, and 49 hips from 40 patients who met the inclusion criteria were included in the study. Demographic data, risk factors, examination findings, comorbidities, age at diagnosis, and age at surgery were evaluated from patient files.

**Figure 1.**
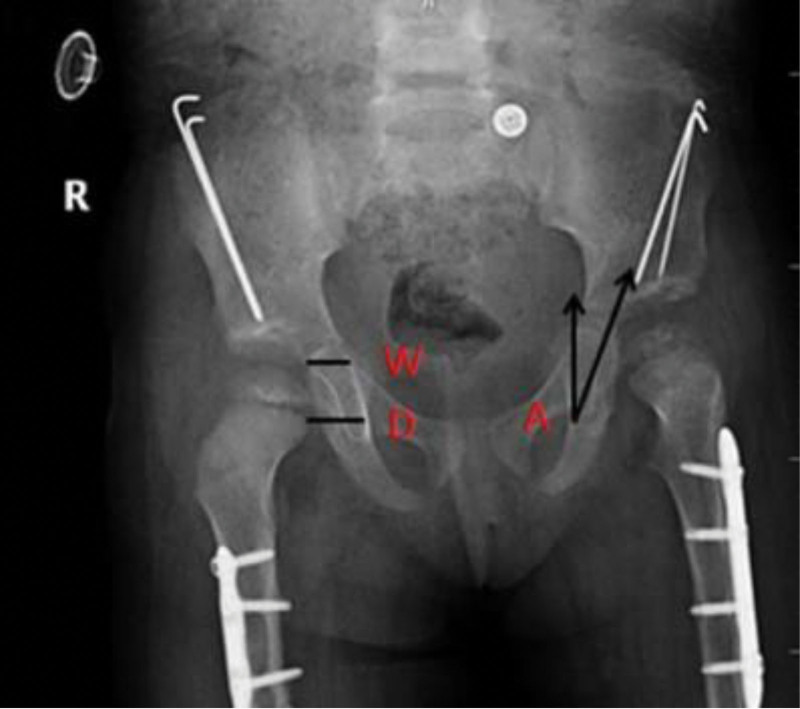
Smith c/b ratio measurement. Pelvic radiograph of a 26-month-old girl who underwent salter innominate osteotomy of her right hip. Measurement of Smith c/b ratio.

**Figure 2.**
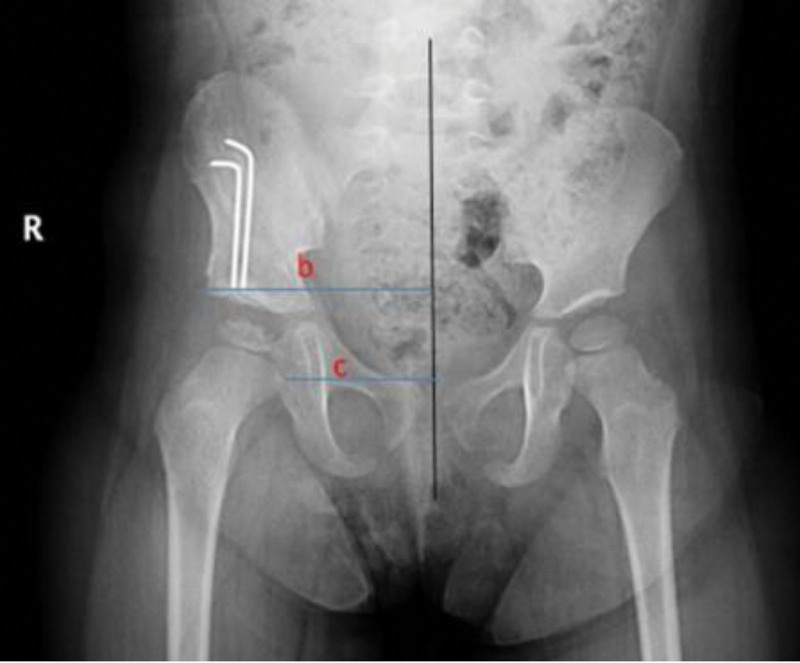
Teardrop figure measurements. Pelvic radiograph of a 25-month-old girl who underwent salter innominate osteotomy of both hips. TDW (W: TDW) and TDD (D: TDD) measurements are shown on the right hip, while ATA (A: ATA) measurement is shown on the left hip. ATA = acetabular teardrop angle, TDD = femoral head teardrop distance, TDW = teardrop width.

Patients were evaluated using preoperative anteroposterior pelvic radiography and divided into 2 groups according to the Tönnis classification. Twenty-five hips of 23 patients with Tönnis stage 2 were included in group 1, and 24 hips of 17 patients with Tönnis stage 3 and Tönnis stage 4 were included in group 2. In addition, the AI, c/b ratio, femoral head teardrop distance (TDD), acetabular teardrop angle (ATA), and teardrop width (TDW) were measured on anteroposterior radiographs of the pelvis (Figs. 1 and 2). Radiological parameters were measured at 4 different time points: preoperative (t1), immediate postoperative (t2), 6-month follow-up (t3), and final follow-up (t4). TDW and ATA measurements were made only at the 6-month follow-up and at the last follow-up, as they are measurements related to the configuration of the teardrop figure. Changes in radiological parameters with respect to time and differences between groups (Groups 1 and 2) were statistically evaluated.

At the last follow-up visit, clinical results were evaluated according to the McKay criteria and radiological results were evaluated according to the Severin evaluation system. The Kalamchi–MacEven criteria were used to evaluate avascular necrosis.

Data were analyzed using the IBM SPSS Statistics 28.0 packet program. The conformity of the data to a normal distribution was examined using the Kolmogorov–Smirnov, Shapiro–Wilk, Skewness and Kurtosis tests. Student *t* test was used to compare normally distributed continuous variables between groups. The relationship between categorical variables was investigated using the chi-square test. The differences between repeated measurements were investigated using repeated measures analysis ANOVA in a linear model. Because there were 2 independent measurements, the sphericity assumption was accepted, and statistical significance was set at *P* < .05.

## 3. Results

The study included 49 hips from40 patients, 37 (92.5%) females and 3 (7.5%) males. Twenty patients had left-sided DDH, 11 had right-sided DDH, and 9 had bilateral DDH. According to Tönnis classification, 25 hips were classified as Tönnis stage 2, 11 as Tönnis stage 3, and 13 as Tönnis stage 4. Tönnis stage 2 hips were included in group 1 and Tönnis stage 3 and 4 hips were included in group 2. Three hips (6%) were previously treated with closed reduction, 4 hips (8%) were treated with pelvic bandage and SIO was performed on the basis of residual acetabular dysplasia, while SIO was performed on the basis of delayed diagnosis and treatment in 42 (86%) hips.

The mean age at surgery was 26.53 (18–53) months. Thirty-four (69%) hips underwent SIO only, whereas15 (31%) underwent SIO with femoral shortening. After a mean follow-up period of 33.7 ± 12.8 months, 38 (78%) hips had excellent results according to the Severin radiologic classification, while 9 (18%) had good, and 4 (4%) had moderate results. Thirty-seven (76%) hips had excellent results according to the McKay clinical classification, 10 (20%) had good results, and 2 (4%) had moderate results. According to the Kalamchi–MacEven criteria, 34 (70%) hips had no radiological evidence of AVN, whereas 10 (20%) hips had stage 1 AVN (Table 1).

**Table 1:** Evaluation of patients in terms of clinical, radiological and femoral head AVN.

	Group 1 (n = 25)	Group 2 (n = 24)	Total (n = 49)	X ^2^	*P* value
Severin classification					
Excellent	19	19	38 (%78)	0.091	.956
Good	5	4	9 (%18)		
Moderate	1	1	2 (%4)		
McCay criteria					
Excellent	19	18	37 (%76)	2.408	.492
Good	4	6	10 (%20)		
Moderate	2	0	2 (%4)		
Kalamchi-MacEwen classification					
Grade 0 (no necrosis)	19	15	34 (%70)	1.051	.591
Grade 1	4	6	10 (%20)		
Grade 2	2	3	5 (%10)		

When Groups 1 and 2 were evaluated separately, no statistically significant difference was found between the groups in terms of clinical, radiological, and femoral head avascular necrosis results (*P* = .591, *P* = 956 and *P* = .492, respectively) (Table 1).

Mean AI angle was 38.4 ± 6.3 degrees preoperatively, 23.7 ± 4.5 degrees in the first follow-up radiograph after surgery, 19.1 ± 4.1 degrees in the 6th month follow-up radiograph, and 16.8 ± 3.9 degrees in the final follow-up. When AI was compared over time (T1, T2, T3, and T4) and between the groups, no significant difference was found; however, a significant difference was found over time (*P* < .001) (Tables 2 and 3).

**Table 2:** Comparison of radiological parameters.

	Group 1 (n = 25)	Group 2 (n = 24)	TOTAL (n = 49)	*P* value
Acetabular index (deg)				
Preoperative	38.77	38.01	38.4	.807
Postoperative 6 mo	19.39	23.50	19.1	
Final follow-up	17.79	15.90	16.8	
c/b ratio				
Preoperative	0.95	1.12	1.01	<.001
Postoperative 6 mo	0.70	0.69	0.70	
Final follow-up	0.66	0.66	0.66	
TDD (mm)				
Preoperative	18.82	24.62	2.07	.02
Postoperative 6 mo	13.15	13.08	13.13	
Final follow-up	11.12	12.15	12.39	
ATA (deg)				
Postoperative 6 mo	14.83	20.83	16.75	.654
Final follow-up	10.64	16.10	12.39	
TDW (mm)				
Postoperative 6 mo	6.48	8.40	7.09	.35
Final follow-up	5.02	5.41	5.14	

**Table 3:** Statistical evaluation of radiologic parameters in different time measurements.

	Preoperative/postoperative 6 mo (*P* value)	Postoperative 6 mo/final follow-up (*P* value)
c/b ratio	1.01/070 (<.001*)	.70/0.66 (<0.001*)
TDD (mm)	20.7/13.13 (<.001*)	13.13/11.45 (*P* = .008*)
ATA (deg)	-	16.75/12.39 (<.001*)
TDW (mm)	-	7.09/5.14 (.001*)
Asetabular indeks (deg)	38.4/19.1 (<.001*)	19.1/16.8 (<.001*)

ATA = acetabular teardrop angle, TDD = femoral head teardrop distance, TDW = teardrop width.

* *P* < .05.

Smith c/b ratio preoperatively averaged 1.01 ± 0.12 mm and postoperatively, 6. The c/b ratio was measured as 0.70 ± 0.05 mm in the monthly control and 0.66 ± 0.05 mm in the last control. A statistically significant difference was found when the c/b ratio was compared over time (T1, T3, and T4) and between the groups (*P* < .001) (Tables 2 and 3).

All patients underwent 3 radiological measurements in relation to the teardrop figure. These were the ATA, TDW, and TDD. Because the teardrop is a physiological structure and its appearance is delayed in hip pathologies, TDW and ATA measurements, which are directly related to the configuration of the teardrop, could not be performed before (t1) and immediately after (t2) surgery. Because the medial border of the teardrop was not affected by hip pathologies, preoperative TDD measurements were possible. TDD was measured preoperatively (t1), at the 6-month follow-up (t3) and at the final follow-up (t4), whereas TDW and ATA were measured at 6th month follow-up (t3) and at the final follow-up (t4). The TDD was was measured as 20.68 ± 4.34 mm preoperatively, 13.13 ± 3.05 mm at the 6-month follow-up, and 11.45 ± 3.24 mm in the final follow-up. TDW was measured as 7.09 ± 2.49 mm at the 6-month follow-up and 5.14 ± 2.28 mm in the last control. ATA was measured 16.75 ± 6.35 degrees in the 6th month control and 12.39 ± 6.49 degrees in the last control. (Table 2). TDD, TDW, and ATA showed statistically significant differences at different time points (*P* < .001). The only parameter that showed a significant difference between Groups 1 and 2 was TDD (*P* = .002) (Table 3).

A significant difference was found between the preoperative and postoperative radiological parameters used in patient the follow-up (*P* < .001). Only the TDD and c/b ratios were significantly different between groups 1 and 2 (*P* = .002 and *P* < .001 respectively) (Table 3).

## 4. Discussion

Many radiological parameters have been defined to evaluate the treatment efficacy and prognosis in patients treated for DDH.^[14,15]^ The most important result obtained in the present study is that ATA, TDW, and TDD, which are measurements related to the teardrop structure, and Smith c/b ratio can be used safely in the determination of treatment efficacy and prognosis.

Teardrop is a “U” shaped physiological structure that can be seen in the anteroposterior radiograph of the pelvis. The medial side of this structure shows the projection of the medial cortex of the posterior acetabulum and the lateral side shows the projection of the lateral aspect of the acetabular fossa. The medial side is unaffected by hip pathologies and is present at birth. The lateral side was absent at birth and visible on radiography after concentric hip reduction. Therefore, the appearance of the teardrop was delayed in patients with a delayed concentric reduction of the hip joint.^[1,14–16]^ In the present study, 32% of the affected hips in Group 1 and 62.5% in Group 2 did not have a teardrop structure preoperatively, and when all patients were evaluated, 46% of the affected hips did not have a teardrop structure preoperatively. In addition, a statistically significant correlation was found between the appearance of the teardrop structure and Tönnis stage (x^2^ = 4573, *P* = .032). In at study conducted by Smith et al^[17]^, teardrop was observed after an average of 6.75 months after stable and successful hip reduction. In our study, 6 hips underwent SIO. For lunar control, at teardrop figure can be observed, and related measurements can be performed.

It has been shown that in all hip pathologies, where concentric reduction of the hip is impaired, the appearance of the teardrop structure is delayed, and its morphology is altered. By examining the morphology of the teardrop structure, hip pathologies, especially DDH, can be treated. The ATA, TDW, and TDD are defined for this purpose.^[12–14,16,17]^

Another radiological parameter is the Smith c/b ratio, which shows lateralization of the femoral head. As it shows minimal changes with age, it can be used safely in the follow-up of patients with DDH in all age groups.^[13]^ In the study by Chang et al^[13]^, the mean preoperative c/b ratio of 63 hips undergoing SIO was 1.11, and an average of 0.71 at the 6th month follow-up. In a study by Gurger et al,^[18]^ the c/b ratio changed significantly postoperatively in patients undergoing SIO. In the study by Akman et al,^[19]^ c/b ratio in patients who underwent SIO was 1.07 preoperatively and 0.72 postoperatively. Li et al examined the change in the c/b ratio after treatment and showed that the c/b ratio was important in determining prognosis. The authors concluded that a c/b ratio that does not decrease significantly may be an important finding for early subluxation and residual dysplasia. Chang et al^[13]^ showed that the c/b ratio is more reliable for determining prognosis than AI and other measurement techniques. The authors reported that 30% of patients with a c/b ratio ≥ 0.72 6 months after surgery and 60% of patients with a c/b ratio ≥ 0.72 1 year after surgery developed residual dysplasia. Based on these findings, c/b ratio can be used as an early prognostic parameter.

AI is the most commonly used radiological parameter in patient follow-up because it measures the severity of dysplasia, acetabular remodeling, and treatment outcomes.^[13,20]^ Studies have reported that AI improves by approximately 10° to 13° with SIO.^[1,21,22]^ In our patients, a mean improvement of 14.6° in the acetabular index was achieved after SIO, which was consistent with the literature.^[1,13,18,21,22]^

In addition to the evaluation of radiological parameters, all patients were evaluated according to the Severin radiological classification, McKay clinical classification, and Kalamchi–MacEven avascular necrosis criteria after a mean follow-up of 33.7 ± 12.8 months. According to the Severin radiological classification, excellent and good results were obtained in 96% of the cases. According to the McKay clinical classification, excellent and good results were obtained in 96% of the cases. According to the Kalamchi–MacEven criteria, 70% of patients had no radiological evidence of AVN, whereas 20% had stage 1 AVN. In a meta-analysis conducted by Merkcaet et al,^[23]^ Severin and McKay scores of 1372 hips were evaluated in 36 studies of patients undergoing SIO. According t Severin classification, excellent and good results were obtained in 84% of cases, whereas according to McKay classification, excellent and good results were obtained in 86.2% of cases. Preoperative Tönnis staging was used in 16 of the 36 studies that were examined. Although there are studies on the clinical and radiological results of Tönnis stage in the literature, these are mostly conservative treatment methods.^[12,24]^ Studies investigating the prognostic relationship between Tönnis stage and clinical and radiological outcomes of patients undergoing pelvic reconstructive procedures are rare.^[12,15,25]^

Consistent with the literature, the clinical and radiological results and avascular necrosis rates obtained in this study is satisfactory.^[12,24–28]^ In addition, results were obtained to support the current evidence revealing that the Tönnis stage had no effect on success in patients undergoing surgical treatment.^[1,8,12,23]^

Radiological parameters other than AI have been used less frequently in literature. Our study showed that the c/b ratio used in the present study is more reliable in determining prognosis than AI and other radiological measurement techniques. Radiological measurements of teardrops are less commonly used. In the present study, the statistically significant changes in ATA, TDW, and TDD measurements over time after SIO showed that these parameters could also be used in patient follow-up.

The small number of patients included in this study and its retrospective design limited our findings. Additionally, the low number of poor clinical and radiological results obtained after SIO has limited the evaluation of radioloical parameters for prognostic monitoring.

## 5. Conclusion

SIO is an effective and successful method used in the late treatment of DDH; however further studies are needed to evaluate the effectiveness of radiological parameters used in the follow-up of patients to determine their prognosis. We believe that multiple radiological parameters should be used in combination with follow-up of patients after SIO and the effectiveness of these parameters in determining prognosis should be investigated with long-term follow-up.

## Acknowledgments

We would like to thank ENAGO (http://www.enago.com.tr) for their contribution in the translation and editing of the manuscript.

## Author contributions

**Conceptualization:** Kadir Ismail Dere, Duran Topak, Okkeş Bilal.

**Data curation:** Kadir Ismail Dere, Duran Topak, Fatih Doğar.

**Formal analysis:** Kadir Ismail Dere, Okkeş Bilal, Burak Kuşcu.

**Funding acquisition:** Kadir Ismail Dere, Fatih Doğar.

**Investigation:** Kadir Ismail Dere, Fatih Doğar.

**Methodology:** Kadir Ismail Dere, Fatih Doğar, Mustafa Abdullah Ozdemir, Burak Kuşcu.

**Project administration:** Kadir Ismail Dere, Burak Kuşcu.

**Resources:** Kadir Ismail Dere.

**Software:** Kadir Ismail Dere.

**Supervision:** Kadir Ismail Dere, Mustafa Abdullah Ozdemir.

**Validation:** Kadir Ismail Dere.

**Visualization:** Kadir Ismail Dere.

**Writing – original draft:** Kadir Ismail Dere.

**Writing – review & editing:** Kadir Ismail Dere, Duran Topak.
